# Health system barriers and levers in implementation of the Expanded Program on Immunization (EPI) in Pakistan: an evidence informed situation analysis

**DOI:** 10.1186/s40985-018-0103-x

**Published:** 2018-09-17

**Authors:** Babar Tasneem Shaikh, Zaeem ul Haq, Nhan Tran, Assad Hafeez

**Affiliations:** 1 Health Services Academy, Chak Shahzad, Park Road, Islamabad, 44000 Pakistan; 2 Johns Hopkins Center for Communication Programs, Islamabad, Pakistan; 3 Alliance for Health Systems and Policy Research, Geneva, Switzerland

**Keywords:** Immunization, Child health, Health system, Pakistan

## Abstract

**Background:**

In Pakistan, immunization coverage has been quite low since the program’s inception, and the 2012–2013 population-based survey recorded it at 54%. Much has been written about the issues, challenges, and constraints in the implementation of Pakistan’s immunization program. However, there is a need to better understand the health system barriers as well as levers that influence progress. This review aims to bridge the information gaps on system-level barriers that currently impede the optimal delivery and uptake of immunization services to the children of Pakistan through the Expanded Program on Immunization (EPI).

**Methods:**

We conducted a comprehensive literature review, using PubMed and Google Scholar to find peer-reviewed literature, and also reviewed EPI-related international and national reports. Additionally, we consulted government reports, surveys, and publications on the health system. Employing the basic tenets of WHO’s health systems framework for health system strengthening, and a socio-ecological model, this study cataloged the service delivery and the demand side perspective on various pillars of Pakistan’s immunization program.

**Results:**

Themes generated from the literature review included financing, governance, service delivery, human resources, information systems, and supplies and vaccines. Findings suggest that certain areas in the larger health system need to be improved for a more coordinated implementation of EPI in Pakistan. Moreover, it is imperative to understand community behaviors and perceptions as well as demand side issues in order to achieve the desired results.

**Conclusion:**

For better immunization coverage and ultimately a reduction in child mortality due to preventable diseases, EPI operations and performance must be improved. Further systematic implementation research could help to develop an even finer understanding of the system-wide bottlenecks encumbering the coverage and efficiency of the program.

## Background

**Fig. 1:**
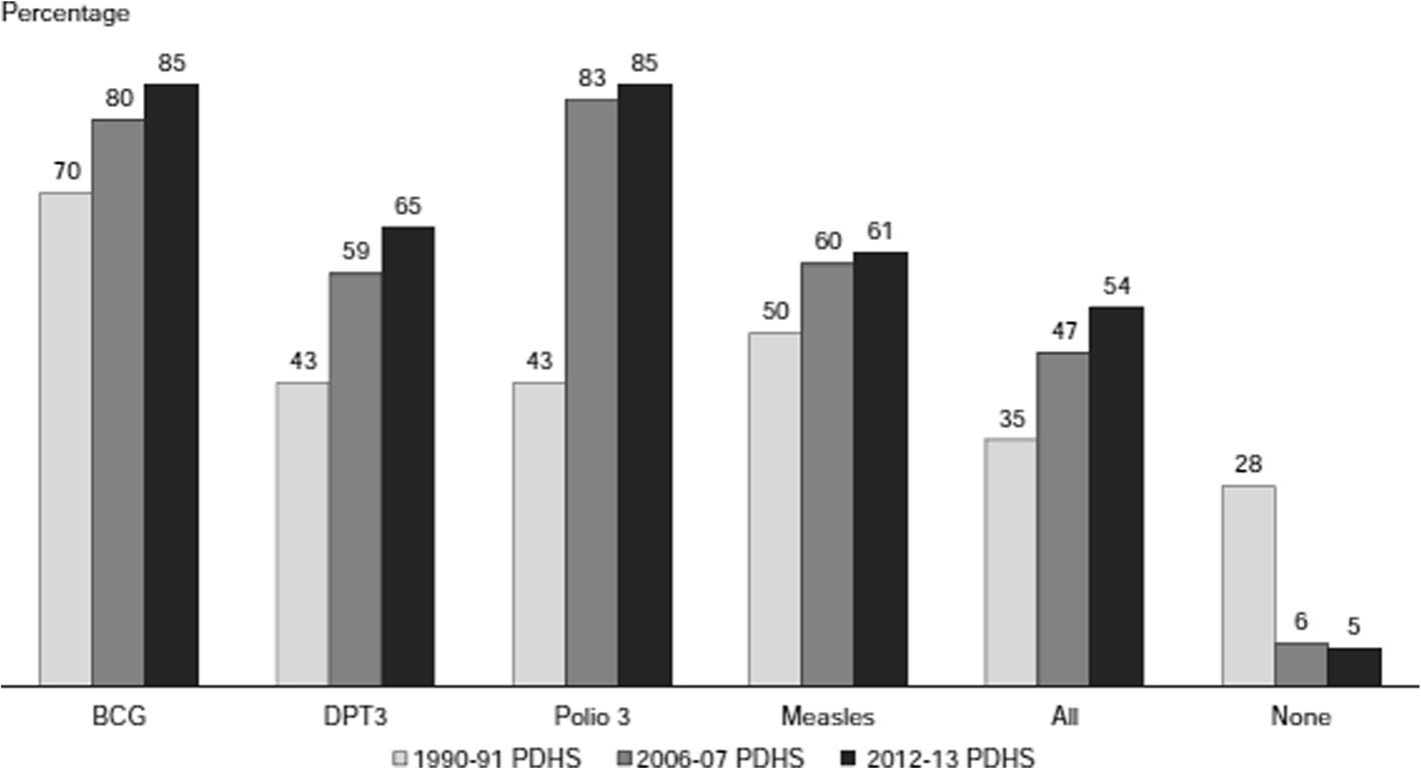
Trends in immunization coverage among children age 12–23 months. Add the source citation [6]

Despite being an established cost-effective public health strategy for improving child survival, each year, millions of children in low- and middle-income countries (LMICs) do not receive the full series of vaccines on their national routine immunization schedule [1, 2]. In Pakistan, over 50% of deaths in post-neonatal children are attributable to pneumonia, diarrhea, or meningitis, which can be prevented through vaccination [3]. The Government of Pakistan initiated the Expanded Program on Immunization (EPI) in 1978 and gradually introduced all requisite antigens, with the recent addition of Rota virus [4]. WHO recommends immunization coverage of 90% at the national level and at least 80% for every district [5]. Pakistan’s immunization indicators have improved since the program’s inception; however, recent data from 2012 to 2013 recorded merely 54% full immunization coverage for children age 12–23 months (Fig. 1) [6].

**Fig. 2:**
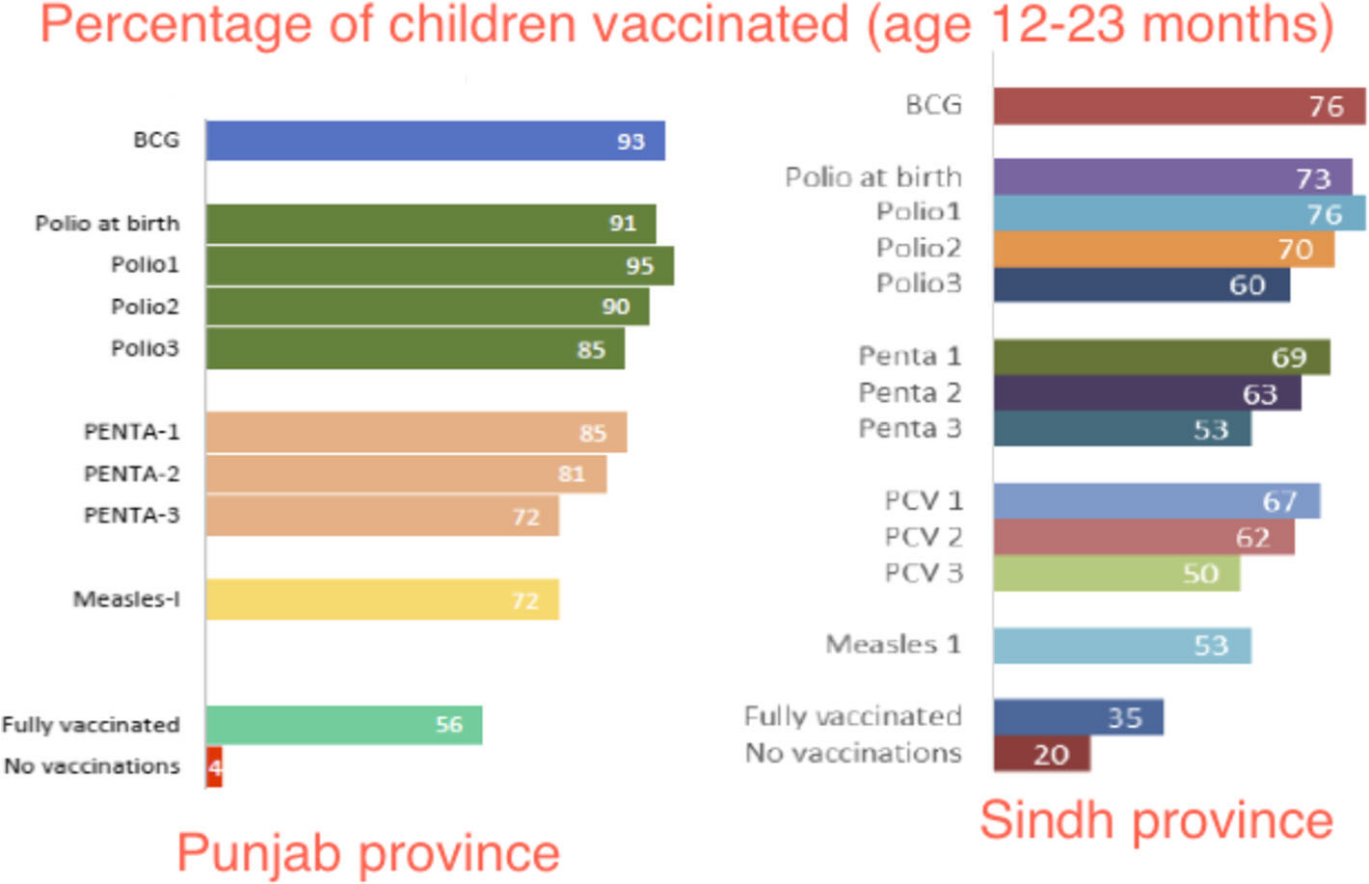
Vaccination coverage in 2014 for children age 12–23 months in Punjab and Sindh provinces. MICS 2014 [7, 8]

Vaccine-specific coverage starting from BCG coverage at 85% falls to 61% for measles (Fig. 1). In addition, there is a large dropout seen from the first two doses of polio (90.2%) and DPT (76.8%) to third doses of the same vaccines (82 and 62.5% respectively). Vaccine coverage drops with birth order; first child coverage is 64%, while only 39% of children born in order 6 or more are fully covered. There are significant regional variations with the Islamabad Capital Territory having the highest percentage (74%), followed by the provinces of Punjab (66%) and Khyber Pakhtunkhwa (53%), whereas immunization coverage is lowest in Sindh province (29%) and Baluchistan province (16%). There are obvious differences in immunization coverage between children of women with no education (40%) and children of literate mothers (74%). Children from households in the highest wealth quintile are much more likely to be fully immunized (75%) as compared to those in the lowest quintile (23%) [6]. In Punjab, the situation seems to be deteriorating (Fig. 2) with the percentage of fully immunized children age 12–23 months dropping to be 56% in 2014 [7], whereas Sindh showed improvement with full immunization coverage increasing to 35% in 2014 [8].

Another national survey from 2014 to 2015 captured a significant gap in the percentage of fully immunized children between rural (56%) and urban (70%) areas. The provincial differences demonstrate similar disparity. The data for urban/rural differences by province were in Sindh (62/33%), Baluchistan (48/20%), Khyber Pakhtunkhwa (74/54%), and Punjab (75/65%). Punjab had the highest immunization rate (70%) followed by Khyber Pakhtunkhwa (58%) and Sindh (45%). Baluchistan, which is the most deprived area, had the lowest coverage with only 27% of children fully immunized [9].

Given this state of affairs, it is evident that there is a need to take stock, particularly to understand the health system-wide barriers as well as the levers that could influence progress and thereon develop strategies to either overcome or capitalize on these factors to optimize performance of the EPI program in Pakistan.

## Methods

This study aims at bridging the information gaps about system-level barriers that currently are impeding the optimal delivery of immunization services to the children of Pakistan. We employed the basic tenets of WHO’s health systems strengthening framework, i.e., governance, financing, service delivery, human resource, information systems, and essential drugs, supplies, and technologies [10], and Sallis’ socio-ecological model which helps in studying the community’s perceptions and behaviors [11]. Hence, this study explored various pillars of the immunization program in Pakistan from both the service delivery and the demand side perspective. We conducted a detailed literature review to document what has been published already about this topic, identified barriers and levers of EPI implementation, and then developed a set of recommendations. Using MeSH terms and key words (Immunization; Child health; Health system; Pakistan), relevant peer-reviewed articles were accessed using PubMed and Google Scholar. Other reports and documents were accessed from the websites of EPI Pakistan and UN agencies. Salient areas emerging from the literature review were cataloged under the building blocks of the health system.

## Results

The information gleaned from the peer-reviewed articles, government reports, EPI documents, WHO/UNICEF/GAVI reports, and some gray literature unravels a stagnant or declining immunization status. At the same time, this analysis also shows a multifactorial picture responsible for the current state of affairs of EPI in Pakistan.

### Program financing

Development partners have always generously supported the maternal, child, and newborn health programs in Pakistan [12]. Although the immunization program mainly depends upon domestic development funds, resources from donors (WHO, UNICEF, GAVI, etc.) have been instrumental too. Pakistan is the biggest recipient of GAVI at present, categorized as a Tier 1 priority country. GAVI financial support to the Pakistan government has been channeled through partner organizations, predominately, WHO and UNICEF [13]. The Government of Pakistan’s own share represents approximately 20% of the total EPI allocations [4]. The Japan International Cooperation Agency (JICA) and the World Bank also support the program. Moreover, the donors have supported in-service trainings for EPI managers. However, delayed release of funds and inefficiencies in expenditure have been noted as some key issues in the past. Of particular note is the lack of appropriation for transportation and fuel costs. Shortage of funds for repair and maintenance of cold chain equipment and vehicles could jeopardize vaccine efficacy [13].

### Program governance

Pakistan went through devolution of its service-related public sectors including health sector with the 18th amendment in its constitution effective from June 28, 2011. The Federal Ministry of Health (MoH) was dissolved, and the overall responsibility for health services policy direction and planning was devolved to the provinces [14]. Inefficiencies and stalled health system’s performance was observed at nearly all of the operational levels for some time after the devolution of 2011 [15]. A lack of clarity in roles and responsibilities of federal and provincial tiers of the government resulted in a vacuum in governance and weak stewardship at decision-making levels. The National Health Vision 2016–2025 later outlined more clearly the roles and responsibilities of the federal and provincial government vis-à-vis health programs and interventions [16]. There is a proposal that going forward, each district must have its own EPI implementation plan, which should consider and address the gaps identified by the situational assessment [13]. Polio in Pakistan has generated much analysis and discussion at the global and national levels. In late 2014, due to a rise in polio cases, an Emergency Operations Centre was established and was mandated to ensure a synergy between the Polio Eradication Initiative and EPI, as well as with other sectors. Nevertheless, this convergence or synergy is still to be seen as fully operational [17]. The role of the private sector as a key stakeholder has also been documented with regard to governance of EPI [18], which could work hand in hand with the public sector in order to achieve the desired targets of immunization, but its potential still remains untapped.

### Human resource

The lack of a comprehensive human resource (HR) strategy has been discussed time and again in the context of EPI in Pakistan. There is no regular and formal training program for the management cadres, and learning is mostly self-directed and on the job. Managers often lack the practical knowledge for leading program operations proficiently [19]. EPI workers’ fatigue due to frequent polio campaigns have reduced their time dedicated to routine EPI vaccination initiatives [13]. In-service training for routine immunization staff is not held on the basis of any planning and programming, rather it is conducted whenever the donor funding is available. Competency of the staff, outreach capacity, service structure, attitudes towards clients, political interference in transfers and postings, and lack of accountability are all notable HR-related issues of EPI [20, 21]. On the other hand, there are workers in the polio program who are willing to perform their duties while putting their lives at risk, and facing extremist sections of the society, amidst a grim law and order situation [22]. Introducing incentive structures among managers and health workers of EPI or contracting with non-governmental organizations (NGOs) can potentially improve the HR performance [23].

### Service delivery

Immunization services provided through outreach are costly and face logistic issues. The outreach strategy of EPI lacks details in micro-plans, has weak monitoring and supervision, and deficient human, operational, and other resources [13]*.* In many rural areas, routine immunization literally comes to naught during National Immunization Days, when all vaccinators are entrusted with the additional responsibility of covering 150–200 children per day, door marking, record keeping in tally sheets, and locating and marking missing children [24]. Coverage of vaccination services requires a rational re-deployment of vaccinators and task shifting to community-based service providers, e.g., lady health workers (LHWs) and community midwives for covering their catchment areas. Vaccinators would thus be able to focus on areas not covered by any workers [25]. Involvement of the private sector and NGO outlets is also one of the solutions, but at present, there is no policy in EPI on formal engagement with the private sector [13].

### Supplies and vaccines

Interrupted supply of vaccines has been reported from time to time. Delays in forecasting, procurement, storage, and distribution to the provinces, districts, and to the “last mile” (i.e., the hardest to reach segments of the population) have suffered in the past because of unduly tedious procedures [13]. Inadequate maintenance of cold chain is another issue reported in the literature. Power outages are frequent, and there is no electricity back up at many places. EPI has state-of-the-art cold chain for vaccine storage and transport; however, its maintenance has been a long-standing issue, particularly in rural remote areas where program monitoring is also weak [26]. Alternative solutions such as solar energy ought to be tried as a backup for power outages.

### Information systems

Unreliable reporting, poor monitoring and supervision systems, and limited use of local data for decision-making are other impediments in the performance of EPI. Data collection is paper-based at the facility level, and then, from district upwards, it becomes electronic. Therefore, establishing its credibility has been a challenge. Moreover, for quite some time, the EPI data was not reflected in the District Health Information System (DHIS) [27]. Inaccurate immunization records lead to the loss of billions of rupees every year [15]. There is a dearth of health systems research to better understand the dynamics between EPI and the beneficiary population [28].

### Community perceptions and behaviors

Low community awareness and misbeliefs that vaccines cause disease and the doubts about vaccine safety and effectiveness have been reported as important factors, impeding the uptake of immunization, especially in the case of polio [29]. Therefore, educating the masses and population segments with low literacy levels, especially the women, is a must for improving the utilization of immunization services [30]. Gender differential in immunization coverage needs innovative gender mainstreaming strategies at the community level such as employing more female vaccinators and community volunteers for outreach to women [31]. Community activists can also encourage people to seek immunization services and can increase demand through educating various community segments [32]. Communication between immunization workers and the parents of children has been flawed, and a positive engagement has helped with overcoming the resistance to vaccinations [33]. On the other hand, service providers in clinics do not emphasize the importance of immunization [34]. Religious beliefs and lack of knowledge about the benefits of the vaccines still dictate many pockets of this highly diverse and populated country [35]. Targeted community awareness programs, a robust surveillance network, and engagement with the dominant religious entities can help to root out the issue [36, 37]. Better understanding of the religion and soliciting local support for vaccination campaigns may assist in negotiating access in the areas where refusal is an issue [38].

Demand side issues and community misperceptions are quite high. Ample funds are allocated for social mobilization, yet meager amounts are spent on communication, and to create community awareness of routine immunization [13]. Moreover, a shift of resources from mass media (TV and radio) to community-level, dialogic communication is proposed, given clear evidence that caregivers rely on healthcare providers, family, and friends for information about immunization [17]. The demand and supply barriers of EPI have been well summarized (Table 1) in an important study undertaken by UNICEF [39].

**Table 1:** Demand and supply side barriers in effective implementation of EPI

Issues	Demand/supply side barriers
1. Low awareness level among caregivers and healthcare providers regarding vaccine-preventable diseases and their risks	Demand
2. Concerns of caregivers about safety of oral polio vaccine	Demand
3. Belief in and use of local remedies for prevention and treatment	Demand and supply
4. Low knowledge and awareness of health care workers regarding VPDs and their prevention	Supply
5. Distance, time, and cost of travel to health facility and long waiting time there	Demand and supply
6. Unavailability of vaccines and vaccinators and dissatisfaction with quality of service	Demand and supply
7. Missing vaccination card in the home	Demand

## Discussion and recommendations

There are several factors which we can bank upon for improving the EPI immunization program in Pakistan: provincial autonomy as called for in the 18th constitutional amendment, re-enactment of a national ministry of health for coordination, the infrastructure needed for the polio program, and the renewed focus of the government and the development partners on routine immunization. No program, however, can improve without looking at it insightfully and searching for the underlying factors that may be the reason for its sub-optimal performance. Our literature review has unraveled some important areas that need further exploration. These areas along with key recommendations are summarized here for future research and to broaden the evidence base for the immunization program in Pakistan and elsewhere.

### Financing and resource allocation

The budgetary allocations, spending, and reporting have to be made more efficient. Switching over to a midterm budgetary framework mode could be a good option for EPI. This mode of financing will be performance-based and target-oriented. Funds must be earmarked for the maintenance of cold chain, which is the most vital component of the entire program.

### Program governance, management, and accountability

The role of the federal ministry of health and federal EPI cell in the overall coordination of immunization services in the country is pivotal. Forums for “interprovincial coordination” and “donor coordination” must be established. Program review meetings held regularly at the federal, provincial, district, and health facility levels may help to improve governance of the program. Involving the private sector can also resolve some governance issues. Furthermore, participation of local organizations, community leaders, and volunteers can provide timely feedback to improve the immunization services.

### Capacity building and human resource

A fresh review and mapping of the EPI HR and their capacity is required for chalking out a plan for an in-service training. This exercise will bring to light the HR gaps at the federal and provincial EPI cells and there lead to recruitment of new vaccinators and women volunteers at the community level. This may help to reduce workload on the existing staff and perhaps task shift to some extent.

### Immunization policy and legislation for service delivery

Private sector, which is the first contact of care seeking for 80% of the population in Pakistan and which is perceived as more trustworthy, must also be engaged for the delivery of routine immunization. This engagement will have the potential to improve access as well as coverage. Likewise, if task shifting to LHWs is required, legislation and policy decisions must be taken expeditiously. The program needs a clear strategy on immunization through outreach as well as fixed centers. Integration of EPI with other public health interventions such as breastfeeding, maternal nutrition, community midwifery, and micronutrients must be considered.

### Information systems

EPI data reliability ought to be enhanced through a critical review of the current reporting system and by objectively examining the procedures, roles, and responsibilities and also the reasons for its under-performance. Employing newer technologies (i.e., GPS, tablets, smart phones, etc.) can potentially improve the timeliness and accuracy of the data.

### Engaging communities

Campaigns for demand creation need careful planning and coordination with communication experts. Increase in the allocation of funds for mass campaigns, and to the districts to customize messages in their local context, is needed. Developing a deeper understanding of locally held perceptions or misperceptions that shape the behaviors of the community will be helpful in certain geographical areas that have historically proved resistant to EPI efforts. Face-to-face communication and advocacy with local opinion leaders and community elders should be continued.

### Risk analysis

Periodic assessment of the high risk, high priority districts and mapping of vulnerable populations must be carried out. Similarly, profiling of HR and logistic gaps is imperative. Timely and correct interpretation of risk analysis is vital for designing context-specific interventions. Community’s role in disease surveillance must also be tapped for early case detection and reporting, initiating an immediate response, and improving outcomes.

## Conclusion

Our literature review unraveled a multifactorial picture responsible for insufficient immunization coverage in Pakistan. Current evidence suggests that focusing on governance of the program, improving facility-based service delivery and addressing community perceptions could result in the biggest payoffs. Within a multi-cultural milieu and a complex health system, the country presents an ideal case for embarking upon more systematic health systems and implementation research to develop an empirical evidence base and to re-build the routine immunization program to serve the people who are most in need. Moreover, university-conducted research must reach implementers. The current situation pleads the case for generating fresh evidence in order to review policy, programmatic approach, service delivery, and stakeholder engagement for improving EPI in Pakistan.

## Abbreviations

BCG: Bacillus Calmette–Guérin
DHIS: District Health Information System
EPI: Expanded Program on Immunization
GAVI: Global Alliance for Vaccines and Immunization
GPS: Global Positioning System
HR: Human resources
JICA: Japan International Cooperation Agency
LHW: Lady health workers
LMICs: Low- and middle-income countries
NGOs: Non-governmental organizations
UNICEF: United Nations Children’s Fund
WHO: World Health Organization
